# Comparison of a novel cholesterol efflux assay using immobilized liposome-bound gel beads with the conventional method

**DOI:** 10.1042/BSR20201495

**Published:** 2020-08-04

**Authors:** Yuna Horiuchi, Shao-Jui Lai, Takahiro Kameda, Minoru Tozuka, Ryunosuke Ohkawa

**Affiliations:** 1 Analytical Laboratory Chemistry, Graduate School of Medical and Dental Sciences, Tokyo Medical and Dental University (TMDU), 1-5-45 Yushima, Bunkyo-ku, Tokyo 113-8519, Japan; 2 Life Science Research Center, Nagano Children’s Hospital, 3100 Toyoshina, Azumino 399-8288, Japan

**Keywords:** apolipoproteinA-I, cholesterol efflux capacity, high-density lipoprotein, immobilized liposome-bound gel beads

## Abstract

Cholesterol efflux capacity (CEC) is an atheroprotective function of high-density lipoprotein (HDL). CEC is currently measured using artificially prepared foam cells composed of cultured macrophage and ^3^H-cholesterol. However, this conventional method is not suitable for clinical laboratory use due to poor repeatability, complexity, and low safety. Recently, we reported a novel CEC assay, called the immobilized liposome-bound gel beads (ILG) method. The ILG method is an alternative to foam cells, comprising gel beads and 4,4-diflioro-4-bora-3a,4a-s-indacene labeled cholesterol (BODIPY-cholesterol) instead of macrophage and ^3^H-cholesterol, respectively. The ILG method has shown adequate basic properties and strong correlation with the conventional method. Here, we aimed to compare this new ILG method with the conventional method in-depth. When apoB-depleted serum was used as the cholesterol acceptor (CA), the ILG method had far better reproducibility than the conventional method. The CEC of major HDL subclasses HDL_2_ and HDL_3_ had similar results in both the ILG and conventional method. However, the ILG method did not reflect the CEC of apolipoprotein (apo) A–I and a minor HDL subclass which uses ATP-binding cassette transporter A1 on foam cells. Superior reproducibility of the ILG method, which is a limitation of the conventional method, and similar CEC results for major HDL subclasses in the ILG and conventional methods, provide further evidence that the ILG method is promising for measuring CEC clinically. However, some HDL subclasses or apo might have poor CEC correlation between these methods. Further research is therefore needed to confirm the clinical significance of estimating CEC by the ILG method.

## Introduction

Cardiovascular disease (CVD) is a leading cause of mortality worldwide [1]. CVD is mainly driven by coronary atherosclerosis, which involves the progressive narrowing of the arteries due to plaque buildup. Since atherosclerosis proceeds asymptomatically, blood tests play important roles in preventing CVD. High-density lipoprotein (HDL) works by suppressing many of the processes involved in atherosclerosis development *in vivo*. Hence, quantification of HDL cholesterol (HDL-C) level in serum has been done for a long time [2]. However, in recent years, some studies reported that HDL-C does not always indicate the CVD risk [3]. This is believed to be due to the heterogeneity of atheroprotective functions among HDL particles. Therefore, studies have been conducted, with a focus on a particular subclass or function of HDL [4,5].

Many HDL functions have already been reported; antioxidation activity on low-density lipoprotein (LDL) oxidation, improvement of endothelial functions, anti-inflammatory property, anti-thrombotic effect and cholesterol efflux capacity (CEC) [6–9]. CEC is the capacity of HDL to pull out cholesterol from foam cells in atherosclerotic lesions. CEC has been actively investigated because some studies showed superior clinical significance for CVD risk evaluation, independent of HDL-C [10,11].

CEC assay is generally performed as follows; first, foam cells are artificially prepared from cultured cells and radioisotope-labeled cholesterol; then, foam cells are incubated with a cholesterol acceptor (CA) which can pick cholesterol up from the foam cells; finally, the radioactivity of the supernatant, including the CA, and of the lysate, are measured to calculate the CEC. In addition to HDL, serum, and apolipoprotein (apo) B-depleted serum (BDS) have been used as the CA [10,12,13]. Although the above method has been commonly used in basic studies, the two main factors which prevent CEC from practical risk estimation are cultured cells and radioisotopes, due to less practicality in clinical laboratory settings.

To solve this problem, we developed a novel CEC method without cultured cells and radioisotopes [14]. In our new method, gel beads were used instead of cultured cells, and liposome containing fluorescently labeled cholesterol instead of radioisotope-labeled cholesterol. This alternative to foam cells was called the immobilized liposome-bound gel beads (ILG). Moreover, BDS was used as the CA in our ILG method because the isolation of HDL from serum is a time-consuming process. BDS is the fraction mainly composed of HDL and serum proteins, and can be separated in a relatively short time by precipitation of apoB-containing lipoproteins by polyethylene glycol (PEG) [15]. Our previous reports have confirmed the usefulness of the ILG method, including stability, correlation with the conventional method, and suitability of the use of BDS [14,16].

Another important assessment of the clinical application of this method is to thoroughly compare with the conventional method. Good correlation of CEC between these two methods has already been confirmed when BDS or whole HDL was used as the CA [15]; however, the evidence is not sufficient. This is because HDL is a group of heterogeneous particles and can be divided into many subclasses based on size, density, charge, and composition of apo and lipids [17]. Furthermore, part of HDL is modified by various substances such as glucose and amino acids *in vivo*. Thus, the distributions of these subclasses vary greatly across individuals and the comparison of CEC measured using each HDL subclass as the CA also needs to be examined prior to assessment of its availability as a clinical test for evaluation of CVD risk in clinical study.

Based on the results of previous studies, further comparisons between the ILG method and conventional method were conducted in the present study, to establish the clinical use of ILG.

## Materials and methods

### Serum samples

Normal serum samples were obtained from healthy volunteers who gave written informed consent at the Tokyo Medical and Dental University, and used as pooled serum. Serum samples were stored at −80°C until use. The present study was approved by our institutional research ethics committee (M2015-546).

### Preparation of BDS

BDS was prepared as described previously [15]. Briefly, 16 μl of 20% PEG (6000 m.w) in 200 mM glycine buffer (pH 7.4), was added to 40 μl of serum, followed by mixing and incubation at room temperature (RT) for 20 min. The mixture was centrifuged at 12000×***g*** for 30 min and the BDS supernatant was isolated.

### Preparation of ILG

ILG was prepared as described previously [16]. Briefly, egg lecithin (10.6 mg) and cholesterol (2.3 mg) were dissolved in 12 ml chloroform, and 30 μl of 0.5 mM 4,4-diflioro-4-bora-3a,4a-s-indacene labeled cholesterol (BODIPY-cholesterol; Avanti Polar Lipids) in chloroform was added to the solution. The lipid film, formed under N_2_ gas, was then dissolved in ether and the solvent was removed by evaporation. After performing this step twice, the lipid film was completely dried under N_2_ gas and suspended in 14 ml of 10 mM Tris/HCl (pH 7.4) containing 150 mM NaCl and 1 mM Na_2_EDTA (Buffer A). Dried Sephacryl S-300 gel beads (0.7 g; GE-Healthcare) were then added to the liposome suspension, followed by swelling for 30 min at RT. The mixture was then treated with seven cycles of freezing (−80°C) and thawing (in water at RT) to induce the formation of large multilamellar vesicles in the Sephacryl S-300 beads. Finally, the gel was sufficiently washed with Buffer A, centrifuged, and resuspended in 10 ml of Buffer A. The gel suspension was stored in the dark at 4°C.

### Cholesterol efflux assay using the ILG method

Cholesterol efflux assay was performed as described previously [14]. Briefly, the ILG was uniformly suspended and the aliquot (100 μl) was dispensed into a 2-ml Eppendorf tube. Then, 150 μl of CA solution (HDL or BDS) or Buffer A was added to the ILG, followed by incubation in the dark at RT for 16 h. Final concentration of the CA solution was adjusted to 20 μg total protein (TP)/ml, 1 mg total cholesterol (TC)/dl (HDL) or 2% as a serum (BDS). CEC measured in each condition is hereafter described as HDL-CEC (TP), HDL-CEC (TC), or BDS-CEC. The mixture was then resuspended by vortexing and centrifugation. The fluorescence of the supernatant was measured (Ex: 485 nm, Em: 538 nm). All samples were assayed in triplicate.

### Cell culture

THP-1, human monocytic cell line, was maintained in RPMI-1640 (Sigma–Aldrich) containing 10% fetal bovine serum, 0.1% penicillin/streptomycin, and 0.1% non-essential amino acids.

### Cholesterol efflux assay using cultured cells (conventional method)

Cholesterol efflux assay using cultured cells was performed as reported previously [18]. Briefly, THP-1 cells were differentiated into macrophages by the stimulation of 100 ng/ml phorbol 12-myristate 13 acetate in RPMI-1640 supplemented with 0.2% bovine serum albumin (BSA) in 24-well cell culture plate at a density of 2.5 × 10^5^ cells/well for 2 days. Then, macrophages were incubated with RPMI-1640 containing 50 µg protein/ml acetylated LDL (acLDL) [19], 1 µCi/ml ^3^H-cholesterol, and 0.2% BSA for 24 h. THP-1 cells were then washed three times with RPMI-1640, followed by equilibration with RPMI-1640 containing 0.2% BSA for 18 h. Cholesterol efflux was assessed in RPMI-1640 medium containing 20 μg TP/ml or 1 mg TC/dl (HDL) or 2% as a serum (BDS) for 4 h. The radioactivity in the medium and cells were determined by scintillation counting. CEC was calculated using the following formula: {^3^H-cholesterol in medium/(^3^H-cholesterol in medium + ^3^H-cholesterol in cells)} × 100. CEC without CA was also calculated with the same formula and subtracted as a non-specific diffusion.

### Isolation of LDL, HDL, HDL_2_ and HDL_3_

LDL (1.019 < d < 1.063 g/dl), HDL (1.063 < d < 1.210 g/ml), HDL_2_ (1.063 < d < 1.125 g/ml) and HDL_3_ (1.125 < d < 1.210 g/dl) were isolated from pooled serum of healthy volunteers by ultracentrifugation as described previously [20]. HDL fraction to isolate apoE-containing HDL as described below was dialyzed against Binding buffer (50 mmol/l NaCl, 5 mmol/l Tris/HCl, pH 7.4); the others were dialyzed against phosphate-buffered saline (PBS). To evaluate whether HDL, HDL_2_ and HDL_3_ were separated, electrophoresis using 8% non-denaturing polyacrylamide gel was carried out, followed by staining with Coomassie Brilliant Blue (CBB).

### Isolation of apoE-containing HDL

ApoE-containing HDL was isolated as in a previous report [21]. Briefly, an aliquot of HDL dialyzed against binding buffer (whole HDL), which was diluted to approximate 0.6 mg protein/ml, was applied into Heparin-Sepharose column (5 ml; GE Healthcare). The unbound fraction was eluted with binding buffer as apoE-deficient HDL. Then, another aliquot of dialyzed HDL was applied into the column and circulated for 1 h. After washing with binding buffer, the bound fraction was eluted with 5 mmol/l Tris/HCl, pH 7.4, containing 1 mol/l NaCl as apoE-containing HDL. Three HDL fractions: apoE-deficient HDL, apoE-containing HDL, and whole HDL were dialyzed against PBS. Confirmation of the isolation of these HDL fractions, sodium dodecyl sulfate/polyacrylamide gel electrophoresis (SDS/PAGE) using 12.5% polyacrylamide gel under non-reducing condition was performed. The separated proteins were electrophoretically transferred to PVDF membrane (Millipore). After blocking with 10 mmol/l Tris-buffered saline (TBS, pH 7.6) containing 5% skim milk, the membrane was incubated with goat anti-apoE polyclonal antibodies in TBS containing 0.5% skim milk. Then, the membrane was incubated with peroxidase-conjugated anti-goat IgG. The bands were visualized using 3,3′-diaminobenzidine tetrahydrochloride (DAB) and hydrogen peroxide.

### *N*-homocysteinylation of HDL

*N*-homocysteinylation of HDL was performed according to a previously reported method with a slight modification [22]. Briefly, dl-homocysteine (Hcy) thiolactone hydrochloride was dissolved in 20 mM Tris/HCl, pH 10.4. The pH was confirmed to be neutral before use, with pH test paper. Approximately 10 mg/ml HDL was mixed with 6 mM Hcy thiolactone solution and incubated at 37°C. Excess Hcy thiolactone was eliminated by dialysis against PBS. To confirm *N*-homocysteinylation of HDL, the samples were incubated with or without cysteamine. Then they were isolated by isoelectric focusing and transferred on to PVDF membranes, followed by visualized with goat anti-apoA–I polyclonal antibodies as primary antibody, peroxidase-conjugated anti-goat IgG as secondary antibody, and DAB and hydrogen peroxide.

### Measurement of protein and lipids

Protein concentration was determined by Lowry’s method. The concentration of TC in HDL was measured using enzymatic test kit (Kyowa Medex Co., Tokyo, Japan).

### Isolation of apoA–I

ApoA–I purification was performed as described previously [23]. Briefly, HDL fraction was delipidated with ethanol/ether 3:2 (v/v) and applied into Sephacryl S-200-HR column, equilibrated with 20 mmol/l Tris/HCl buffer, pH 7.4, containing 6.8 mol/l urea. The isolated lipid-free apoA–I was dialyzed against PBS and stored at −20°C until use.

### Statistical analysis

Comparisons of TP/TC ratio and CEC between the HDL subclasses were performed using paired *t* tests. Differences in CEC among the apoA–I or HDL of different concentrations were evaluated by repeated-measures using ANOVA with Bonferroni correction. The results are expressed as means ± SD. *P*<0.05 was considered statistically significant. Individual *P*-values are included in the text and figures.

## Results

### Intra- and inter-assay repeatability of the ILG and conventional methods

**Figure 1:**
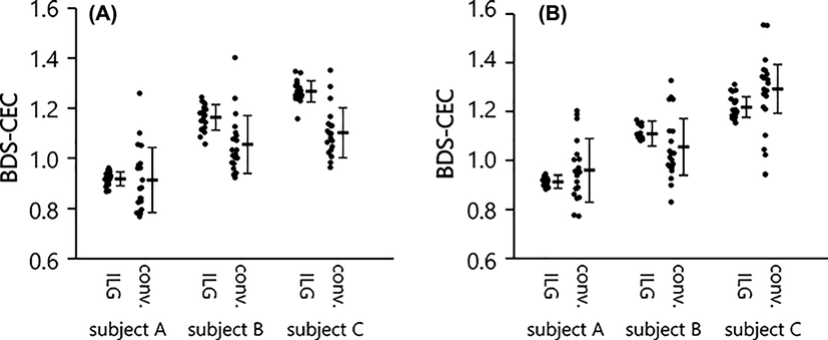
Repeatability of the conventional and ILG methods CEC was measured using four BDSs by the ILG and conventional (conv.) methods. The three results (subject A, B, and C) were normalized by the result of the other BDS and presented as means ± SD. (**A**) Intra-assay repeatability was evaluated by measuring the same samples 20 times simultaneously. (**B**) Inter-assay repeatability was evaluated by measuring the samples in triplicate in 20 measurements.

To evaluate the intra- and inter-assay repeatability of the two types of CEC methods, the CEC of four BDSs were measured. CEC of each BDS was determined 20-times simultaneously for intra-assay repeatability, and in triplicate each day for 20 days for inter-assay repeatability. The results of each BDS were normalized to the results of a reference BDS, and described as subjects A, B and C. Intra-assay reproducibility of the ILG method were less than 5% CV (2.92, 4.44 and 3.34% for A, B and C), while those of the conventional method were approximately 10% CV or more (14.17, 10.98 and 9.11% for A, B and C) (Figure 1A). Inter-assay repeatability of the ILG method was also far superior than the conventional method; the former was less than 5% CV and the latter was more than 15% (3.30, 3.43, and 4.74% for A, B and C in the ILG method, and 18.83, 16.55, and 16.96% for A, B and C in the conventional method) (Figure 1B).

### CEC of HDL_2_ and HDL_3_

**Figure 2:**
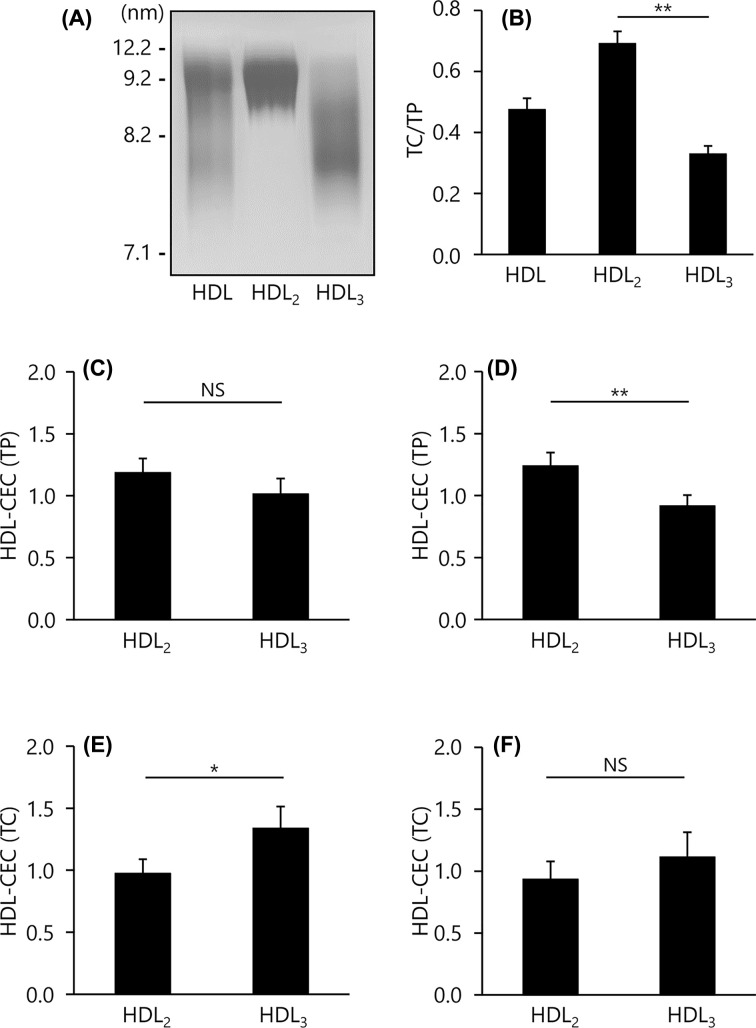
CEC of HDL_2_ and HDL_3_ (**A**) HDL subfractions (5 μg TP/lane) were separated by non-denaturing PAGE, followed by visualization with CBB. (**B**) The content of TC in whole HDL (HDL), HDL_2_, and HDL_3_ were determined as ratios relative to TP content. (**C**–**F**) CEC was evaluated using 20 μg TP/ml or 1 mg TC/dl of each HDL subclasses by the ILG (C,E) and conventional (D,F) method. The results were indicated as the ratio to CEC of whole HDL. All samples were assayed in triplicate. Values are presented as means ± SD (*n*=4). *P<0.05, **P<0.01 by paired *t* tests. Abbreviation: NS, no significance.

To confirm the purification of HDL_2_ and HDL_3_, particle sizes, cholesterol and protein levels in both fractions were examined. Native-PAGE profile showed that HDL_2_ was mainly distributed in the 9.2–12.2 nm interval and HDL_3_ was distributed in <9.2 nm as described previously [24] (Figure 2A). Consistent with previous reports [25,26], HDL_2_ contained more TC than HDL_3_ (TC/TP ratio = 0.69 vs. 0.33, respectively); *P*<0.01 (Figure 2B). Using these HDL subfractions, CEC values were compared. HDL–CEC (TP) of HDL_2_ was higher than that of HDL_3_ by both the ILG and conventional methods; HDL_2_ (1.19 ± 0.11, *P*=0.076, and 1.24 ± 0.11, *P*<0.05, respectively) *vs.* HDL_3_ (1.01 ± 0.12, and 0.92 ± 0.09, respectively) (Figure 2C,D). In contrast, HDL-CEC (TC) of HDL_2_ was lower that of HDL_3_ in both methods; HDL_2_ (0.97 ± 0.11, *P*<0.01, and 0.93 ± 0.15, *P*=0.096, respectively) *vs.* HDL_3_ (1.34 ± 0.17 and 1.11 ± 0.20, respectively) (Figure 2E,F).

### CEC of apoE-containing HDL and apoE-deficient HDL

**Figure 3:**
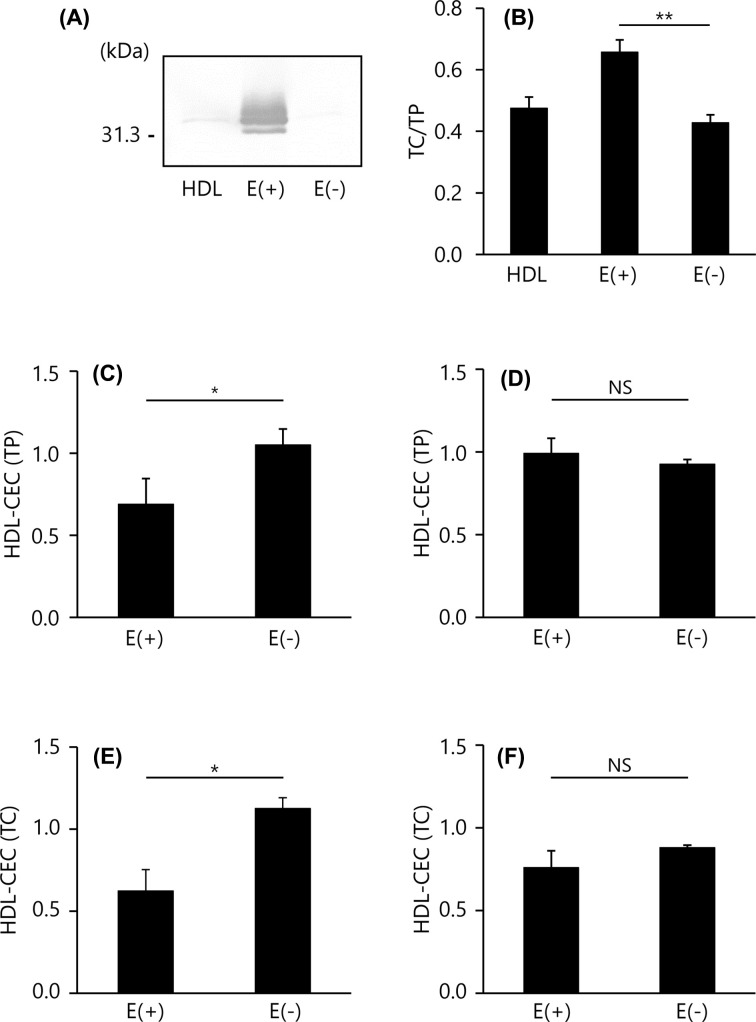
CEC of apoE-containing HDL and apoE-deficient HDL (**A**) ApoE content in HDL subfractions were determined by SDS/PAGE and Western blotting (0.2 μg TP/lane). (**B**) The content of TC in whole HDL (HDL), apoE-containing HDL (E(+)) and apoE-deficient HDL (E(−)) were determined as ratios relative to TP content. (**C–F**) CEC was evaluated using 20 μg TP/ml or 1 mg TC/dl of each HDL subclass by the ILG (C,E) and conventional (D,F) method. The results were indicated as the ratio to CEC of whole HDL. All samples were assayed in triplicate. Values are presented as means ± SD (*n*=3). *P<0.05 by paired *t* tests. Abbreviation: NS, no significance.

Next, we compared the CEC values between the two methods using a minor but well-studied HDL, apoE-containing HDL. Applying an HDL sample to a heparin-Sepharose column allows the separation of apoE-containing HDL and apoE-deficient HDL as shown in Figure 3A. In addition, apoE-containing HDL had more cholesterol (TC/TP ratio = 0.66) than apoE-deficient HDL (TC/TP ratio = 0.43), *P*<0.01 (Figure 3B). CEC values of apoE-containing and -deficient HDL were compared. Both the HDL-CEC (TP) and HDL-CEC (TC) of apoE-containing HDL were significantly lower than that of apoE-deficient HDL in the ILG method (0.68 ± 0.16 vs. 1.05 ± 0.10, *P*<0.05 in HDL-CEC (TP) and 0.62 ± 0.13 vs. 1.13 ± 0.07, *P*<0.05 in HDL-CEC (TC), respectively) (Figure 3C,E). However, there were no differences in the conventional method (0.99 ± 0.10 vs. 0.93 ± 0.03, *P*=0.120 in HDL-CEC (TP) and 0.76 ± 0.10 vs 0.88 ± 0.02, *P*=0.104 in HDL-CEC (TC), respectively) (Figure 3D,F).

### CEC of N-homocysteinylated HDL

**Figure 4:**
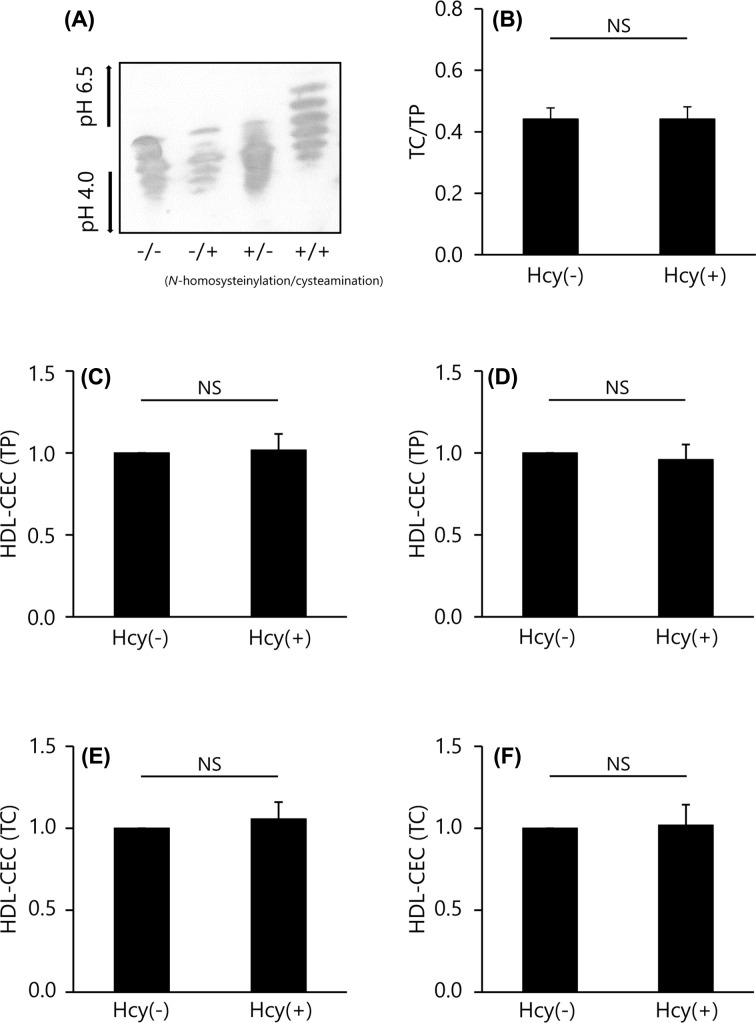
CEC of *N*-Hcy-HDL (**A**) *N-*homocysteinylation of HDL was confirmed by isoelectric focusing and Western blotting (1 μg TP/lane) after treatment with or without cysteamine. (**B**) The content of TC in intact HDL (Hcy(−)) and *N*-Hcy-HDL (Hcy(+)) were determined as ratios relative to TP content. (**C–F**) CEC was evaluated using 20 μg TP/ml or 1 mg TC/dl of each HDL subclass by ILG (C,E) and conventional (D,F) method. The results were indicated as the ratio to CEC of intact HDL. All samples were assayed in triplicate. Values are presented as means ± SD (*n*=4). The results were evaluated by paired *t* tests. Abbreviation: NS, no significance.

Hcy thiolactone was reacted with normal HDL, resulting in the formation of an amide bond with the ε-amino group in lysine residues of the surface apoA–I to produce the *N*-homocysteinylated HDL (*N*-Hcy-HDL). When the *N*-Hcy-HDL was treated with cysteamine, isoelectric profiling (IEF) showed several bands of apoA–I isoform at positions of higher isoelectric point (pI) values, indicating that cysteamine bound to the cysteine residues of homocysteinylated apoA–I (Figure 4A). Conversely, HDL treated with either Hcy thiolactone or cysteamine had a similar pattern to untreated HDL as described previously (Figure 4A) [27]. For lipid and protein composition, as expected, there was no difference in TC/TP ratio of intact and *N*-Hcy-HDL (TC/TP ratio = 0.44 and 0.44, respectively, *P*=0.991) (Figure 4B). Using the *N*-Hcy-HDL, CEC values were compared between the conventional and ILG methods. The CEC of *N*-Hcy-HDL per protein and per cholesterol both showed no difference from intact HDL regardless of the method (1.02 ± 0.10 and 1.06 ± 0.10 times higher than the CEC of intact HDL in the ILG method (HDL-CEC (TP and TC, respectively) (Figure 4C,E); and 0.96 ± 0.09 and 1.02 ± 0.13 times higher than CEC of intact HDL in the conventional method (HDL-CEC (TP and TC, respectively) (Figure 4D,F).

### CEC of apoA–I

**Figure 5:**
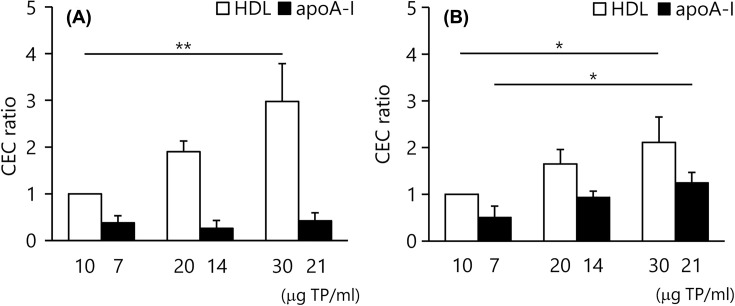
CEC of apoA–I CEC of HDL (10, 20 and 30 μg TP/ml, open bar) and apoA–I (7, 14 and 21 μg TP/ml, closed bar) were determined by the ILG (**A**) and conventional (**B**) methods. The results were indicated as the ratio to CEC of 10 μg HDL. All samples were assayed in triplicate. Values are presented as means ± SD (*n*=3) *P<0.05 and **P<0.01 by repeated-measures ANOVA with Bonferroni correction. Abbreviation: ANOVA, analysis of variance.

For both the ILG method and conventional method, we further investigated the dose-dependency of the CA (HDL or lipid-free apoA–I) for CEC. CEC of HDL by both methods significantly increased with the amount of CA; 1.90 ± 0.23 (20 mg TP/ml) and 2.98 ± 0.81 (30 mg TP/ml, *P*<0.01) times higher than CEC of 10 mg TP/ml HDL in the ILG method (Figure 5A, open bar), and 1.65 ± 0.31 (20 mg TP/mL) and 2.11 ± 0.54 (30 mg TP/ml, *P*<0.05) times higher than CEC of 10 mg TP/ml HDL in the conventional method (Figure 5B, open bar). However, CEC of apoA–I in the ILG method showed no change, whereas it increased like HDL in the conventional method. The relative CEC values of 7, 14 and 21 mg TP/ml apoA–I to 10 mg TP/ml HDL in the ILG method and conventional method were 0.38 ± 0.15, 0.26 ± 0.17 and 0.42 ± 0.18 (Figure 5A, closed bar), and 0.51 ± 0.24, 0.94 ± 0.13 and 1.25 ± 0.22 (Figure 5B, closed bar), respectively.

## Discussion

The CEC assay using ILG was developed as an alternative for the conventional CEC assay which uses cultured cells. We previously reported that CECs measured by the ILG and conventional methods using THP-1 cells were well correlated [14]. However, more detailed investigation was needed to apply the ILG method in a clinical laboratory setting. The present study aimed to compare the ILG and conventional methods more in-depth.

At first, intra- and inter-assay repeatabilities were compared between the two methods. Poor repeatability in the conventional method is one of the factors limiting CEC evaluation in the clinical setting. As expected, repeatability of CEC measured by the conventional method was inadequate to be measured clinically (10–20% CV). In contrast, the ILG method had enough repeatability within 5% CV inferring that the ILG method improved the deficiencies of the conventional method.

It has been reported that CECs measured by the conventional and ILG methods, correlated well when BDS or whole HDL fraction was used as the CA [14]. However, HDL is a group of heterogeneous particles which have different compositions of lipids or apo. A detailed comparison is required to use the ILG method in clinical practice because the composition of these particles is individual dependent. In the present study, CEC was compared using three types of HDL subclasses: major, minor and modified HDL.

HDL_2_ and HDL_3_ are thought to be the most major HDL subclasses. First, we evaluated whether the CEC of these subclasses matched between both methods. HDL_2_ and HDL_3_ had obviously different TC/TP ratio and particle size. These results were consistent with previous reports [25,26] and indicated that HDL_2_ and HDL_3_ were well separated. In both methods, CEC of these HDL showed similar results; HDL-CEC (TP) and HDL-CEC (TC) of HDL_2_ were higher and lower than those of HDL_3_, respectively. The opposite results of HDL_2_ with higher HDL-CEC (TP) and lower HDL-CEC (TC) than HDL_3_ are attributed to the difference in TC/TP ratio. These corresponding results in the CEC of major subclasses suggest that the ILG method can be more successfully used instead of the conventional method in evaluating CVD risk.

ApoE-containing HDL is one of the minor HDL subclasses which accounts for up to 10% of the total HDL [28]. We previously reported that cholesterol efflux is performed through the ATP-binding cassette transporter A1 (ABCA1) in THP-1 cells [21]. In the present study, the CEC of apoE-containing HDL by ILG and conventional methods were compared with that of the other part of HDL, apoE-deficient HDL. Consistent with a previous report [21], apoE-containing HDL had a higher TC/TP ratio. The results of Western blotting indicated that apoE-containing and -deficient HDL were well separated. Although these HDL subclasses have obviously different TC/TP ratios, both HDL-CEC of apoE-containing HDL were significantly lower than those of apoE-deficient HDL in the ILG method despite the fact that there were no differences between CECs in the conventional method. According to these results, it is presumed that the ILG method cannot measure a part of the CEC of apoE-containing HDL measured in the conventional method.

HDL is known to be modified by some enzymes or substances in atherosclerotic lesions [29]. Hcy, an amino acid with a sulfhydryl group, is known as an independent risk factor for atherosclerosis and the concentration is correlated with the severity of CVD [30,31]. Hcy binds and modifies many types of proteins, including apoA–I in HDL [32]. Therefore, *N*-Hcy-HDL is thought to have some key roles in the progression of atherosclerosis. According to a previous report, we confirmed that *N*-homocysteinylation of HDL was successfully produced by Hcy thiolactone [22]. The ILG and conventional methods showed the same results; there were no differences between the CEC of intact and *N*-Hcy-HDL.

To summarize the above results for HDL-CECs, the CEC of some HDL subclasses did not correlate between the conventional and ILG methods. We hypothesized that this was because ILG does not have cholesterol transporters, which are expressed on foam cells. Three transporters, ABCA1, ABCG1 and scavenger receptor class B type I (SR-BI), are well known to be used in cholesterol efflux [33]. Most HDLs pull out cholesterol from foam cells using ABCG1 and SR-BI [33]. In contrast, as described above, we previously reported that apoE-containing HDL uses ABCA1 for cholesterol efflux [21]. Considering these results, it is presumed that the ILG method did not reflect efflux via ABCA1 in foam cell.

To confirm this assumption, CEC of apoA–I was measured by both methods and compared. ApoA–I, a dominant protein of HDL, is well known to pull out cholesterol from foam cells via ABCA1 [33]. As expected, CEC of HDL significantly increased in a concentration-dependent manner in both methods. As apoA–I accounts for approximately 70% of protein in HDL, CEC of apoA–I, with 70% of HDL as protein, were compared with the corresponding CEC of HDL. In the conventional method, CEC of apoA–I significantly increased like that of HDL. However, there was no such increase in the ILG method. This result raised the possibility that the ILG method does not reflect CEC via ABCA1 in THP-1 cell.

ApoA–I and apoE-containing HDL, however, are found in minute amounts *in vivo*. The impact of that difference on CVD risk evaluation might be negligible [28]. Alternatively, some reports elucidated the clinical significance of CEC using foam cells whose ABCA1 transporter was overexpressed by a reagent such as cyclic-AMP (cAMP) [10,11]. On the basis of these reports, it is apparent that CEC via ABCA1 transporter has a great impact on CVD risk evaluation. In addition, cAMP-induced CEC showed better association with CVD risk than total CEC from cAMP-treated cells [34]. Thus, when CEC is measured with purpose of investigation for the potential effects of genetic manipulation/drug on macrophages and their effects on CEC, such as modulating ABCA1 and ABCG1, the conventional assay is useful instead of ILG method using gel beads with lack of these transporters, while ILG has still advantages enough to clinical settings as mentioned above. Above all, both foam cells and ILG are simply tools for evaluating the function of HDL and there is not enough evidence of transporter expression in atherosclerotic lesions. Therefore, it is difficult to conclude whether the different cholesterol efflux mechanisms between the ILG and conventional methods have obvious effects on the risk evaluation without further investigation in CVD patients.

In conclusion, in addition to its simplicity and suitability for clinical use, the present study revealed that the ILG method is superior to the conventional method in repeatability. Furthermore, CEC by the ILG method was thought to generally reflect that of the conventional method according to consistent results in the major HDL subclasses, HDL_2_ and HDL_3_. However, by the difference of principles between the ILG and conventional methods, ILG method has some limitations such that it does not reflect CEC of lipid-free apoA–I and may not be suitable for studies to elucidate detail cholesterol efflux mechanisms *in vivo*. Although the ILG method might be a valuable tool for better predicting CVD risk, further studies such as the investigation of the clinical significance of CEC measured by ILG method are needed to validate the use of CEC in clinical practice.

## Abbreviations

ABCA1: ATP-binding cassette A1
apo: apolipoprotein
BDS: apo B-depleted serum
BSA: bovine serum albumin
cAMP: cyclic-AMP
CA: cholesterol acceptor
CEC: cholesterol efflux capacity
CVD: cardiovascular disease
DAB: 3,3′-diaminobenzidine tetrahydrochloride
Hcy: homocysteine
HDL: high-density lipoprotein
HDL-C: HDL cholesterol
ILG: immobilized liposome-bound gel bead
LDL: low-density lipoprotein
*N*-Hcy-HDL: N-homocysteinylated HDL
PBS: phosphate-buffered saline
PEG: polyethylene glycol
RT: room temperature
SR-BI: scavenger receptor class B type I
TBS: Tris-buffered saline
TC: total cholesterol
TP: total protein
